# Venezuelan equine encephalitis virus: the problem is not over for tropical America

**DOI:** 10.1186/s12941-020-00360-4

**Published:** 2020-05-19

**Authors:** Camilo Guzmán-Terán, Alfonso Calderón-Rangel, Alfonso Rodriguez-Morales, Salim Mattar

**Affiliations:** 1 Instituto de Investigaciones Biológicas del Trópico (IIBT), Programa Regencia en Farmacia, Facultad de Ciencias de la Salud, Universidad de Córdoba, Montería, Córdoba Colombia; 2 Instituto de Investigaciones Biológicas del Trópico (IIBT), Facultad de Medicina Veterinaria y Zootecnia, Universidad de Córdoba, Montería, Córdoba Colombia; 3 Public Health and Infection Research Group, Faculty of Health Sciences, Universidad Tecnológica de Pereira, Pereira, Risaralda Colombia; 4 Grupo de Investigación Biomedicina, Faculty of Medicine, Fundación Universitaria Autónoma de las Américas, Pereira, Risaralda Colombia

**Keywords:** Arbovirus, Equine, Chiroptera, Alphavirus, Zoonoses, Americas

## Abstract

The equine encephalitis viruses, Venezuelan (VEEV), East (EEEV) and West (WEEV), belong to the genus alphavirus, family Togaviridae and still represent a threat for human and animal public health in the Americas. In both, these infections are characterized by high viremia, rash, fever, encephalitis and death. VEEV encephalitis is similar, clinically, to other arboviral diseases, such as dengue, Zika or chikungunya. Most of the alphaviruses are transmitted between vertebrates and mosquitoes. They are able to replicate in a wide number of hosts, including mammals, birds, reptiles, amphibian and arthropods. The VEEV has enzootic and epizootic transmission cycles. At the enzootic one, enzootic strains (subtype I, serotypes D–F and serotypes II–VI) are continuously circulating between mosquitoes and wild rodents in tropical forests and mangroves of the Americas. The main reseroivrs are wild rodent species of the subfamily *Sigmodontinae*. However, bats can be also accidental reservoirs of VEEV. In this article, we reviewed the main features, epidemiology, clinical aspects and the current perspectives of the VEEV.

## Background

Venezuelan equine encephalomyelitis is caused by the Venezuelan equine encephalitis virus (VEEV). Mosquito vectors transmit the virus, mainly affects Equidae, humans, and wild animals. The enzootic cycle of VEEV is complex and infects many different species of mammals and mosquitoes. Some of the enzootic viral species within the Alphavirus genus are known as VEEV, Mosso das Pedras virus, Everglades virus, Mucambo virus, Tonate virus, Pixuna virus, Cabassou virus, and Río Negro virus [1].

The spread of VEEV varies in speed and intensity according to the viral subtype and the densities of mosquito populations, and the transmission is produced by the vector that feeds on an infected animal and subsequently infects a new host. The epizootic subtypes of VEEV are amplified in Equidae, where they spread rapidly and can be highly pathogenic in horses, donkeys, and mules. The disease in horses is characterized by fever, loss of appetite, and disorders of the central nervous system, such as muscle deterioration, blindness, and seizures. In humans, early symptoms of VEEV infection include flu-like symptoms, such as fever, chills, malaise, severe headache, myalgia in the legs and lower back, tachycardia, and in some cases, nausea, vomiting, and diarrhea. If there are neurological signs, they can include seizures, drowsiness, confusion, and photophobia. Children are more likely to suffer permanent neurological damage. After 4 to 6 days of acute illness, the patient may feel weak for several weeks. Lethal cases manifest with diffuse congestion and edema in the brain, gastrointestinal tract, and pulmonary hemorrhage and sometimes triggers meningoencephalitis.

### Biological characteristics of VEEV

The International Virus Taxonomy Committee classified the virus into the Togaviridae family, genus Alphavirus, and according to the Baltimore classification: Group IV (+) ssRNA (REF). The VEEV has single-stranded RNA approximately 70 nm in diameter, with icosahedral symmetry. The 5′ end of the genome encodes four non-structural proteins, nsP1, nsP2, nsP3 and nsP4, and the 3′ end is responsible for three structural proteins, the capsid and the envelope proteins E1 and E2. Non-structural proteins participate in the replication of the viral genome involved in functions of the cytoplasm of the host.

### VEEV and other similar equine encephalitis in America

**Fig. 1:**
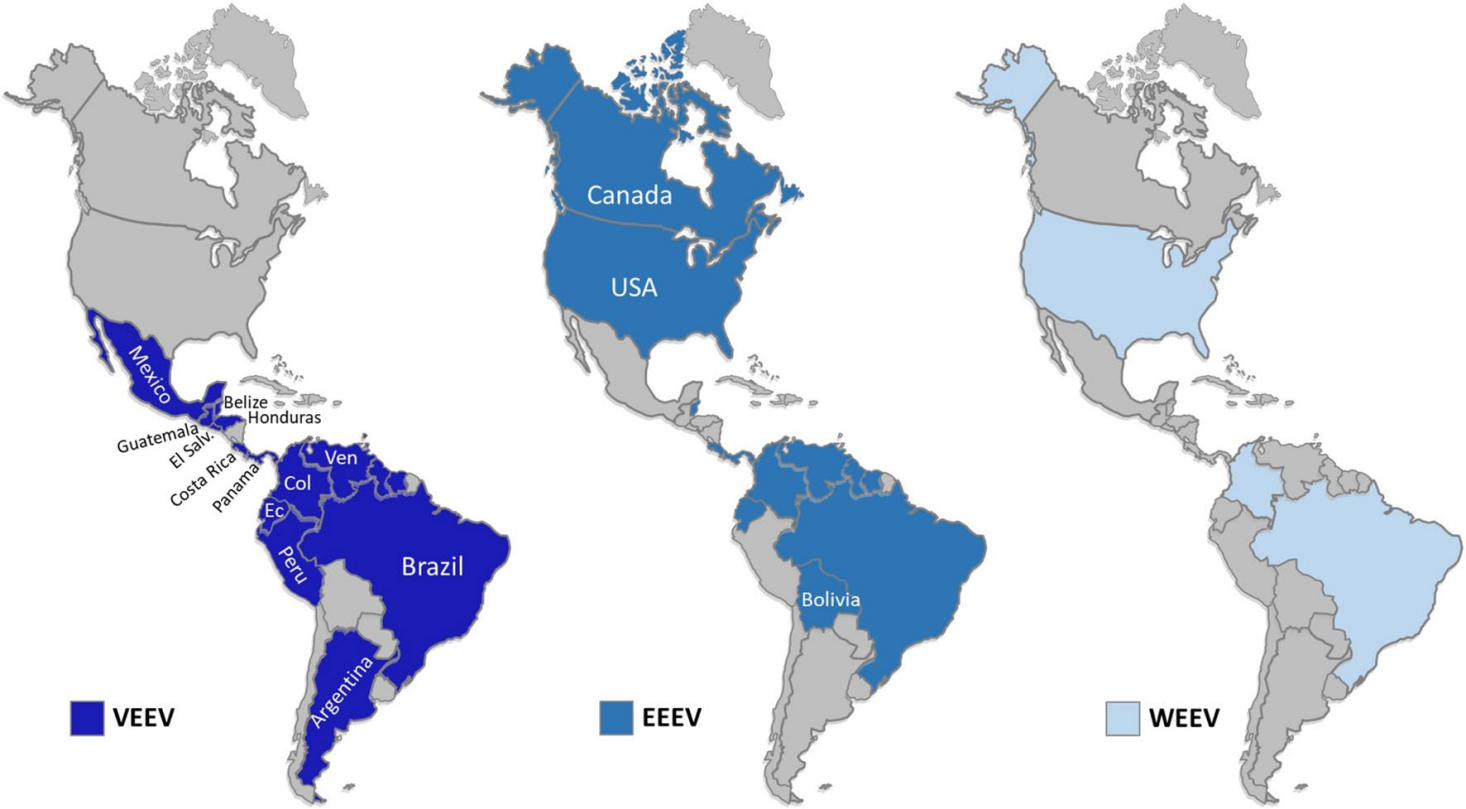
Geographical distribution of the equine encephalitis in the Americas. **a** VEEV. **b** EEEV. **c** WEEV

In addition to VEEV, Eastern equine encephalitis (EEEV), Western (WEEV) are frequent viral infections in the Americas (Fig. 1), which also belong to the genus Alphavirus (*Togaviridae*). In humans, WEEV, EEEV viruses, like VEEV, cause symptoms ranging from mild febrile illness to severe encephalitis that can lead to death [2]. As a result of vaccination, in some countries such as the United States, there are no longer periodically severe epidemics of EEEV and WEEV encephalitis (Fig. 1). However, sporadic cases and small outbreaks are observed. The VEEV continues to present epidemic outbreaks in South America in equine and human populations [3, 4].

WEEV is a natural chimera resulting from the recombination of the EEEV virus and the Sindbis virus (SINV) [5]. It circulates in North and South America among especially passerine birds and *Culex tarsalis* mosquitoes its main vector, mammals can participate in a second cycle [4, 6]. Between 1930 and 1950, WEEV produced widespread outbreaks that covered western North America, extending northward into Saskatchewan, Canada [7], the epizootic reached the east side of the Canadian Rockies [8, 9]. The incidence of WEEV has decreased over the past four decades [10], the last human case in North America occurred in 1994, and the virus has not been detected in mosquito pools since 2008. The WEEV was isolated in Argentina in 2009. Subsequently, an outbreak in Uruguay resulted in a fatal human case [11] (Fig. 1).

With respect, the North American variant of the EEEV is found in Eastern Canada, and in all states east of the Mississippi, it has also been isolated in Arkansas, Minnesota, South Dakota, and Texas. The South American variant is found in areas of Central and South America and along the coast of the Gulf of Mexico. The numerous strains of the EEEV can be grouped into two variants. The variant found in North America is more pathogenic than the variant of Central and South America. Most of the Caribbean strains belong to the North American EEEV group, but the South American variant can also be found (Fig. 1).

Due to genetic divergence and significant differences in ecology and pathogenesis, the isolations of South America from EEEV were recently classified as a distinct species that was called Madariaga virus (MADV) [12, 13]. However, unlike the EEEV of North America, the MADV, formerly known as equine encephalitis of the South American East, was not associated with outbreaks in humans before 2010 when the first outbreak of the MADV was reported in the Darien region of Panama. Before the Darién outbreak, in South America, there were only three reported cases of EEEV in humans in Brazil and Trinidad. Unlike the epidemiological profile in South America, in North America, eight human cases of neuroinvasive EEEV disease have been reported on average between 2004 and 2013 [12, 13]. From the epidemiological perspective, it is interesting to observe that when comparing the epidemics and epizootics of MADV and VEEV, the latter are explosive and involve equine amplification, which has resulted in 100,000 cases or more in humans and thousands of equine deaths in Latin America.

### Epidemiology of Venezuelan equine encephalitis

**Fig. 2:**
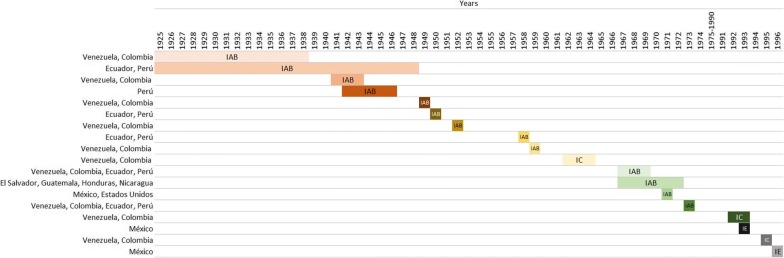
Time evolution of the epizootics of VEEV in the Americas [14, 25, 38, 47–54.]

The clinical manifestation of the disease was described as a “mad plague” in South America in the 1920s [14]. The VEEV was isolated in 1938 from a horse that died of encephalitis [1, 14, 15]. Subsequently, the virus was isolated in 1950 from humans during an outbreak of the disease in Espinal, Tolima, in southern Colombia. The virus is widely distributed throughout the Americas, and outbreaks have been reported in Venezuela, Colombia, Ecuador, Panama, Costa Rica, Nicaragua Guatemala, Honduras, Salvador, Panama, Mexico and the United States [1]. The VEEV has enzootic and epizootic serotypes. Within the group of alphaviruses that cause encephalitis in Equidae and humans are the EEEV and WEEV, Mayaro, Mucambo, and Everglades viruses [16, 17]. Encephalitis caused by VEEV is an emerging infectious disease in Latin America [1, 18]. Outbreaks have been documented for decades in countries with enzootic circulation. The implementation of surveillance systems has allowed the detection of additional human cases in countries and areas with the previously unknown activity of VEEV. Clinically, VEEV is indistinguishable from dengue and other arbovirus diseases, and confirmatory diagnosis requires specialized laboratory tests that are difficult to implement in regions with limited resources. Therefore, an endemic disease in developing countries remains mostly unknown. Surveillance suggests that it may represent up to 10% of the burden of dengue in neo-tropical cities, or tens of thousands of cases per year throughout Latin America [19] (Fig. 2).

### Relationship of the antigenic subgroups and the epidemiology of VEEV

The essential antigenic subgroups of VEEV are subtype (I), which also includes five subtypes of different origins: lA (Venezuela and Trinidad), IB (Peru and Argentina), IC (Venezuela and Colombia), ID (Colombia and Panama), lE (Panama and Mexico); Subtype (II) Florida; Subtype (III) Mucambo. Isolated in Brazil, Suriname, and Trinidad; Subtype (IV) Pixuna. Isolated so far only in Brazil; Subtype (V) Cabassou. Isolated in French Guiana mosquitoes; Subtype (VI). AG8O-663. Isolated in mosquitoes from Argentina [18, 20] (Table 1).

**Table 1:** Subtypes and serotypes of the VEEV complex

Subtypes	Species	Serotypes	Transmission cycles
I	VEE virus	AB	Epizootic
	VEE virus	C	
	VEE virus	D	Enzootic
	VEE virus	E	
	Mosso das Pedras virus	F	
II	Everglades virus		
III	Mucambo virus	A	
	Tonate virus	B	
	Mucambo virus	C	
	Mucambo virus	D	
IV	Pixuna		
V	Cabassou virus		
VI	Rio Negro virus		

Epidemic/epizootic types include the IAB and IC subtypes, both being responsible for significant disease outbreaks in humans and horses. The other subtypes or enzootic viruses such as type ID cause disease in humans and usually do not affect horses [18, 20]. Horses are susceptible to the virus, and the lethality rate for horses is between 20 and 80% [21]. VEEV is neurotropic and neuroinvasive, the virus replicates in lymphoid tissue and the brain, causing an acute viral infection that is typically resolved by the innate and adaptive response. However, neurological symptoms occur in 14% of human infections, and approximately 1% result in lethal encephalitis.

Humans have a minor role in the epidemiology of epizootic cycles because the horses have a greater area exposed to mosquitoes. For this reason, they are considered as natural amplifiers of the disease, capable of generating large viremia and infecting new vectors, which can transmit the disease. Epizootic viruses tend to spread to areas where susceptible animals are found, and this phenomenon makes it easy to spread when favorable conditions are found [19].

Concerning enzootic cycles in which, without the participation of horses, it occurs when new reservoirs enter an enzootic cycle such as small rodents or birds, which can amplify and perpetuate the cycle and affect humans. This process can not only be explained by the exit of the virus from its normal cycle, but there may be factors intrinsic to the virus that increases its virulence [17]. Enzootic cycles are found in defined geographical areas and tropical climates with rainy seasons, near water sources such as swamps. These small cycles are maintained thanks to the mosquito—rodent—mosquito interaction. The antigenic subtypes ID, IE, II, III, and IV, are considered enzootic [22].

### Vectors and hosts

The enzootic subtypes of VEEV are frequently detected and isolated in ecological habitats, where they circulate in cycles of transmission between rodents and mosquitoes. The main reservoirs are sigmodontine rodents, wild species of *Oryzomys, Zigodontomys, Heteromys*, *Peromyscus*, and *Proechimys*. These animals become infected in nature and develop viremia that is sufficient to infect vectors [23, 24].

Enzootic transmission cycles have been described for the varieties ID, IE, Everglades, Mucambo, and Tonate (Bijou Bridge); all of which, with the exception of the Bijou Bridge, are transmitted and maintained in a cycle involving rodents and mosquitoes of the subgenus *Culex* (*Melanoconion*) [25–27]. The Bijou bridge virus is transmitted by the insect, *Oeciacus vicarius*, to birds in western North America [28].

Enzootic cycles have also been inferred for the Pixuna and Río Negro viruses and involve *Culex* (*Melanoconion*), *Aedes, Psorophora*, and other mosquitoes [27, 29]. It is believed that the *Culex* (*Melanoconion*) species are the primary vectors of most or all enzootic complex strains of VEEV [29]. VEEV in bats of America. In 1970 in Oxaca, Mexico was found a vampire bat *D. rotundus* infected with an epidemic strain of VEEV [1]. In Guatemala, a serological study was carried out on 939 Neotropic bats of 22 species of an enzootic focus of VEEV 1971-1975, and antibodies against VEEV were found in seven species of bats, three belonging to the genus *Artibeus*, the virus was isolated from *Uroderma bilobatum*. This study suggested that the genus *Artibeus* is regularly infected with the virus and that it may serve as an alternative host for virus maintenance [15].

In Trinidad and Tobago, a seroprevalence study was carried out against antibodies selected from flavivirus and alphavirus in a sample of 384 bats. The sera were analyzed by ELISA using specific antibodies against West Nile virus (VWN), VEEV, EEEV. 2.9% of the sample (11/384) were seropositive against VEEV-specific antibodies, none of the sera were seropositive against VWN and EEEV virus antibodies [30]. A study conducted in the Montes Azules reserve in Chiapas, Mexico, on 146 bats and 14 rodents, molecular evidence of VEEV was obtained in bats of the species *Artibeus lituratus*, *Carollia sowelli, Glossophaga sorici*na and *Sturnira parvidens* and in rodents (*Sigmodon hispidus* and *Oryzomys alfaroi*). Rodent specimens were negative for VEEV, WEEV, and WNV [31]. A study carried out in the Colombian Caribbean area, 286 bats were captured, VEEV was detected in the fruit species *Artibeus planirostris* and *Sturnira lilium*. Immunohistochemistry with monoclonal antibodies confirmed the presence of VEEV in the brain, spleens, and lungs of fruit bats. However, in the liver, heart, and kidney, the virus was not found.

### Pathophysiology of VEEV

The virus is transmitted by the bite of mosquitoes, which is infected by feeding on reservoirs that contain high levels of viremia. The viremia process is produced in the salivary glands, and then, the viruses’ passage to new reservoirs during the feeding of the vector. The mosquito saliva is deposited in the extravascular space of the affected vertebrae, then by the interaction of the E2 protein and receptors on the surface of the host cell, the viruses enter the cell by endocytosis and are taken to endosomes. The low pH at endosomes releases components of the virus, they move to the cytoplasm through pores in the endosome. The nucleocapsid is disassembled in the cytoplasm, leading to virus replication [32]. Many species of mosquitoes can transmit this virus because the high epidemic power allows it to infect and produce many cases in humans and animals in a short time. Among the species capable of transmitting VEEV are Culex (Melaconion), Aedes, Mansonia, Psorophora, Haemagogus, Sabethes, Deinocerites, and Anopheles. It is also possible that some classes of Diptera may transmit the virus.

The *Culex* species is the one that maintains the transmission of enzootic viruses in nature, while a wide range of mosquitoes transmits epizootic viruses. The disease is almost clinically indistinguishable from other viral diseases such as dengue or influenza, and in reality, several epidemics of Venezuelan equine encephalitis have been diagnosed in their initial stages as dengue. Usually, the disease begins suddenly with severe headache, fever, chills, myalgia, retro-ocular pain, nausea, and vomiting. Infections in 80% are mild, and last only 3 to 5 days. In many cases, the febrile course is diphasic, after a few days of fever there may be signs that affect the central nervous system, ranging from drowsiness to encephalitis with disorientation, seizures, coma, and death paralysis. The involvement of the central nervous system is more frequent in children; it is estimated that about 5% of those under 15 who become infected with VEEV can develop neurological conditions. However, in children under 5 years, this figure can be increased to 35%. In severe cases, the proportion of lethality can be as high as 10%, in many cases, there may be sequelae such as mental incapacity, epilepsy, learning difficulties, hydrocephalus, personality changes, and paralysis. A study conducted by London et al. in-Rhesus monkeys showed that VEEV infection is a risk to the fetus, capable of causing congenital disabilities, fetal death, and abortion. VEEV has a severe teratogenic effect on the central nervous system of primates. Viral titers obtained from the brain, uterus, and other organs indicated that fetal infection and virus replication occurred. Congenital microcephaly, hydrocephalus, and cataracts in 67% of the animals were found [33].

In pregnant women who have suffered VEEV infection, an increase in abortions and births of children with congenital malformations has been found, especially at the level of the central nervous system [34]. The mechanism by which VEEV invades the central nervous system is not completely clear. In hamsters, it has been documented that the invasion occurs through olfactory neurons when the inoculation is nasal or by aerosols. However, this mechanism has also been observed after peripheral inoculation. Natural immunity to the disease is mediated by the presence of antibodies against the E2 glycoprotein. These antibodies are protective and persist throughout life. One of the factors that seem to be more critical within the virulence of the enzootic and epizootic strains of VEEV is the presence of early elimination of the infecting virions. In less virulent strains, this elimination occurs in less than 30 min in experimentally infected animals, while more virulent strains take much longer to be removed. Other essential characteristics of less virulent strains are that they induce minor viremias, and the titers found in lymphoid organs are smaller than those of more virulent strains. Currently, there are no vaccines for VEEV in humans; however, two vaccines are used under the research status of new drugs to protect laboratory personnel. The live attenuated TC-83 strain vaccine protects animals from subcutaneous and aerosol exposure, and 82% of humans develop immunity after vaccination [21, 35].

### Epizootics and epidemics

The first well-documented outbreak involving equines occurred in Valle del Cauca, Colombia, in 1935 and dispersed to Venezuela the following year. In 1943 the outbreak reached Trinidad (Table 2) [36]. Additional epizootics were reported in Peru from 1942 to 1946 [25, 37]. One of the most massive outbreaks of the VEEV started in Guajira Colombia, in 1962, initially involved about 3000 human cases, of which 20 were fatal, this outbreak then expanded to Venezuela where 23,283 human cases were presented including 960 neurological cases and 156 deaths reported in 26 months (Fig. 2; Table 2).

**Table 2:** VEEV outbreaks in animals and humans in the Americas, 1935–2011

Year	Country	Equines	Humans
1935	Colombia	Unknown	–
1935–1943	Colombia	Unknown	–
1935–1943	Venezuela	Unknown	–
1942–1946	Peru	Unknown	–
1943	Trinidad	Unknown	–
1962	Colombia	Unknown	3000
1962	Venezuela	Unknown	23,283
1967–1968	Colombia	Unknown	Unknown
1967–1968	Venezuela	Unknown	Unknown
1969	Ecuador	20,000	31,000
1969	El Salvador	Unknown	Unknown
1969	Guatemala	Unknown	Unknown
1969	Mexico	50,000	Unknown
1969	Other CA countries	Unknown	Unknown
1971	USA	2000	110
1972	Mexico	Unknown	–
1992	Venezuela	Unknown	Unknown
1993	Venezuela	Unknown	Unknown
1993	Mexico	Unknown	–
1995	Venezuela	4000	100,000
	Colombia		
1996	Mexico	Unknown	–
1998	Colombia	Unknown	250
2011	Colombia	4 foci	–
2011	Venezuela	3 foci	–

During 1967 and 1968, epizootics occurred in Colombia, but the exact number of cases in horses and humans was not reported. In early 1969, a massive outbreak was reported in Ecuador, in which approximately 31,000 cases involved 310 deaths and approximately 20,000 equine deaths (Fig. 2; Table 2).

At the end of 1969, epizootics were reported in El Salvador and Guatemala; these outbreaks eventually spread to most of Central America and Mexico [38–40]. During this outbreak, about 50,000 horses died, in addition to about 52,000 human cases, of which 93 were fatal in Mexico [41, 42]. Initially, the deaths of horses in Mexico were reported in the state of Chiapas, this outbreak then spread northward in 17 Mexican states, following the path of susceptible Equidae, to the Gulf coast and, finally, to southern Texas [42, 43]. The outbreak was finally contained when more than 8 million doses of the TC-83 vaccine were applied to the equines; in addition, vector control was implemented [42] (Fig. 2; Table 2).

The last cases of Mexican equines were recorded in September 1972 in the Marias Islands [43]. In Texas, between June and August 1971, about 2,000 infected horses were reported, including 1426 associated deaths. During the same period, 110 human cases were confirmed. After 19 years of inactivity, VEEV outbreaks were again reported in the Americas. In 1992, a first outbreak was reported in Venezuela, followed by additional outbreaks in 1993 in both Venezuela and Mexico [40] (Fig. 2; Table 2).

In 1995, both Venezuela and Colombia reported outbreaks [44] involving some 100,000 human cases, of which 3000 suffered neurological complications, with 300 associated deaths [44]. There were also at least 4000 equine deaths associated with this outbreak. Small equine epizootics were also reported in Mexico in 1993 and 1996 [40] (Fig. 2; Table 2).

The most recent Colombian VEEV epidemic occurred in the department of Chocó in the period between February and March 2008. Approximately 13% (250/2000) of the inhabitants had a febrile illness compatible with VEEV; two died but could not Isolate the virus during the outbreak. In Colombia, during 2011, there were four foci of encephalitis in horses, three foci of Venezuelan equine encephalitis in the departments of Córdoba and Magdalena, and a focus of eastern equine encephalitis in the department of Casanare (Table 2). Additionally, there was a case of VEEV confirmed by seroconversion in a minor in the municipality of Barrancabermeja, bordering Venezuela. Venezuelan equine encephalitis has an epidemiological behavior of persistence with apparent epidemiological silences. However, this is because, in countries endemic to flaviviruses, the first diagnostic impression is Dengue and recently Zika. Despite having had an epidemic peak of 40,000 cases by 1995, over time, the number of human cases has significantly decreased, and cases in animals have sporadically occurred. In Colombia, in the last 17 years, there has been a significant reduction in the foci of Venezuelan equine encephalitis. However, in 2010, there was an increase attributable to risk factors such as humid tropical areas, with vector proliferation, flooding of the Magdalena, San Jorge and Sinú rivers, and high rainfall of 800–1200 mm. Associated with this, Equidae used as working animals on farms are not vaccinated, which allows viral amplification [45].

### Novel strategies for prevention and control

Vector control is the foremost approach accessible for controlling several VBDs, including VEEV [46]. For other diseases are vaccines available for human use, such as is the case of yellow fever, Japanese encephalitis, and dengue. But for VEEV only vaccines for animals are available. Then, vector control is currently the only method available to protect human people. Vector control seeks to limit the transmission of pathogens by reducing or eliminating human contact with the vectors. A broad range of vector control tools exists, which can be broadly classified into chemical- and not–chemical-based tools. Means pursuing immature vectors can act by destroying the immature stages (e.g., chemical or biological larvicides and predator species) or by removing viable aquatic habitats (e.g., habitat modification or manipulation). Means oriented to the adult vectors function by destroying the vector (e.g., indoor residual spraying [IRS], space spraying) and reducing vector contact (blood-feeding success) with human and animal reservoir hosts (e.g., topical repellents, house screening, insecticide-treated bed nets [ITNs], insecticide-treated dog collars). There are also several novel vector control tools under development, e.g., genetic manipulation of mosquitoes, a bacterial infection of vectors (e.g., Wolbachia), and insecticide-treated eave tubes. The most essential novel strategies, still to be appropriately implemented in VEEV, are gene drive, *Wolbachia*, spatial repellents, and eave tubes [46].

Gene drive is an approach of genetic alteration that can be used to spread favorable traits through interbreeding populations of malaria mosquitoes, still to be proved on *Culex*. *Wolbachia* is a bacteria genus that naturally infect certain insect species, not typically found in mosquitoes. That has not yet assessed in the VEEV vectors, although effectively on *Aedes*. Spatial repellents are compounds that prevent vectors from entering spaces occupied by a potential human host to reduce encounters between the vector and the host. Eave tubes are small plastic tubes with insecticide-laden electrostatic netting that are inserted into the house wall, below the roof. Mosquitoes are lured to the house by host outdoors emanating through the eave tubes and are killed after contacting the insecticide-treated netting [46].

## Conclusions

In conclusion, Venezuelan equine encephalitis is a persistent zoonosis in Latin America through long periods of apparent epidemiological silence. Rodents can be their main reservoirs; however, there are no robust eco-epidemiological studies that demonstrate their role in zoonosis. Bats could have a potential role as dispersing hosts for this virus. Equine surveillance is useful as a predictive indicator or sentinel to prevent cases in humans.

## Data Availability

Not applicable.
